# Details of Medical Inspection

**Published:** 1908-02-22

**Authors:** 


### Public Health and Hygiene.

### DETAILS OF MEDICAL INSPECTION.

The Schedule and Circular (582) issued by the Board of Education on January 23, in response to requests for further and more definite guidance as regards the details of the work of medical inspection, will do much to simplify the task of the medical advisers of local education authorities.

The chief difficulties to be considered are administrative rather than educational or scientific. There is comparatively little dispute as to the end in view, or as to the means which, from the technical standpoint of medical science and practice, should be adopted for its complete attainment.

The board are fully aware of these difficulties, and in preparing their memorandum and regulations it was necessary for them to consider what system would best reconcile the theoretical and practical considerations, and overcome the divergence between the ultimate end and the end immediately attainable, or between the methods which are scientifically desirable and those which can be applied in existing circumstances at the initiation of the work under the Act.

In the accompanying schedule the board indicate the particulars, attention to which they regard as constituting the minimum of efficient medical inspection, and they consider that at least these particulars should be included in any other schedule which the Local Education Authority may authorise for use in their schools. It deliberately excludes many points of anthropometric or statistical interest which are worthy of attention, and which it is hoped may receive attention in suitable districts. Nor does it profess to lay down the lines of a clinical study or of a scientifically complete medical examination. It is intended to indicate the methods which, in the board's opinion, should be followed and the particulars which should be attended to for the purpose of determining the fitness of the individual child for school life, to guide the authority in adapting education to the peculiarities or abnormalities of the child, and to prepare the way for measures for the amelioration of defects in the child or its environment.

A more elaborate and complete form could readily be devised, but the board's knowledge of the circumstances in which the work is to be done leads them to believe that greater elaboration would in the majority of cases defeat its own end.

Accompanying the Schedule are specimen cards submitted as samples of the ways in which the records may be made and kept by the officers.

The Board of Education is to be congratulated on suggesting the adoption of modern methods of record making and keeping. Nothing is so admirably adapted to the needs of this new work as the method known as the card system. Many medical officers of health have already adopted it in their departments, and have found it a vast improvement on the older system of keeping their records in books, which entails an immense amount of unnecessary clerical work in duplicating entries. The specimen cards submitted by the Board of Education are obviously suggestions to the local medical officers as to the form which it is open to them to adapt to their own special requirements. It is clearly of great importance that some uniform standard of schedule, of size of card, and of periodicity of inspection should be established, the more so in that it is intended that the record of the results of medical inspection made at one school should follow it to another should the child remove during its school life to another district. We think it would have been better on this account not to have suggested alternative sizes of cards, but to have recommended a uniform standard of size. The 5 in. by 8 in. card has been found by experience to be as large as can readily be manipulated for purposes of abstracting and tabulating, and if this size were universally adopted, the card sent from one medical officer to another would take its place in the file without the need of the entries being copied on to a new card. It suggests itself that it would be well to adopt uniform symbols for recording the observations of the medical officer, and this can only be secured upon the initiative of the Board.

We think also a better quality of card than the one submitted will prove not only economical but necessary. The cards will be handled so frequently during the child's school life that, unless they are proof against fracture on rough handling, there will be not only the cost of a duplicate card, but also the much greater cost of doubling the clerical work. These, of course, are details which must be left to the initiative of the local authority.

We should have liked to see a definite recommendation of the exclusive use of the metric system for measurements. The work being new, a fair start on this basis would have precluded the difficulties and complications which will almost necessarily follow upon the employment of both metric and English measurements. The employment of both, however, is much better than the alternative use of either, and we do not doubt that ultimately the metric system will come into exclusive use in all anthropometric observations. The spacing of the different headings of the Schedule will doubtless undergo modification in the hands of different medical officers bent on observations in excess of the minimum in respect of this or that item of the Board's Schedule.

Taken altogether, the suggestions in regard to detail made by the Board are admirable. The difficult task of leaving local initiative elastic while securing the broad minimum of uniformity essential for national comparative results has been well overcome. The framework for a national survey of the physical condition of the children, capable of adjustment or expansion to meet particular ends and needs has been laid down. At the same time a uniform observance of the necessarily stereotyped minimum will yield a mass of statistics on the vital conditions of the children, the value of which as future data for social structure and reform it is difficult to overestimate. We do not doubt that this medical survey heralds the most profound advances in State medicine.

